# Enjoying Sad Music: Paradox or Parallel Processes?

**DOI:** 10.3389/fnhum.2016.00312

**Published:** 2016-06-24

**Authors:** Emery Schubert

**Affiliations:** Empirical Musicology Laboratory, School of the Arts and Media, University of New South WalesSydney, NSW, Australia

**Keywords:** sadness, hedonic tone, music, aesthetic experience, context, dissociation, negative emotion

## Abstract

Enjoyment of negative emotions in music is seen by many as a paradox. This article argues that the paradox exists because it is difficult to view the process that generates enjoyment as being part of the same system that also generates the subjective negative feeling. Compensation theories explain the paradox as the compensation of a negative emotion by the concomitant presence of one or more positive emotions. But compensation brings us no closer to explaining the paradox because it does not explain how experiencing sadness itself is enjoyed. The solution proposed is that an emotion is determined by three critical processes—labeled motivational action tendency (MAT), subjective feeling (SF) and Appraisal. For many emotions the MAT and SF processes are coupled in valence. For example, happiness has positive MAT and positive SF, annoyance has negative MAT and negative SF. However, it is argued that in an aesthetic context, such as listening to music, emotion processes can become decoupled. The decoupling is controlled by the Appraisal process, which can assess if the context of the sadness is real-life (where coupling occurs) or aesthetic (where decoupling can occur). In an aesthetic context sadness retains its negative SF but the aversive, negative MAT is inhibited, leaving sadness to still be experienced as a negative valanced emotion, while contributing to the overall positive MAT. Individual differences, mood and previous experiences mediate the degree to which the aversive aspects of MAT are inhibited according to this Parallel Processing Hypothesis (PPH). The reason for hesitancy in considering or testing PPH, as well as the preponderance of research on sadness at the exclusion of other negative emotions, are discussed.

Enjoyment of negative emotion in music is seen by many researchers and lay persons as a paradox. In recent years there has been a small boom of interest in addressing the question empirically, with a range of explanations including: (1) that the phenomenon is proportionally rare compared to other emotional responses to music, making it an overemphasized phenomenon; and (2) that there is some additional aspect of the music that is liked, which compensates for the sadness, making the apparent enjoyment of the negative emotion illusory (for an overview, see Eerola et al., 2015). Along these lines, there is evidence that we in fact experience a mix of emotions, positive such as happiness along with negative ones such as sadness (Hunter et al., 2008, 2010) or a wide range of emotions, which may happen to include sadness (for a summary, see Eerola et al., 2015), or that we are actually observing the negative emotion in the music, rather than fully experiencing it (Kawakami et al., 2013). But each of these views more or less brush aside the conservative estimate that 25% of people report enjoyment of sadness and other negative emotions in response to music (Schubert, 2010, 2013b; Huron, 2011). And it must be emphasized that these participants claim to “feel” sad, not just observe it in the music (Huron, 2011; Schubert, 2013a). It is this group of participants who present the interesting problem to be addressed: actually feeling a negative emotion and enjoying that feeling (Garrido and Schubert, 2013, 2015).

In the above list of explanations, little attention has been given to the possibility that enjoyment and feeling sadness each belong to different cognitive processes that comprise the emotion of sadness while operating in parallel. However, the idea of processes operating in parallel could provide a parsimonious explanation of the paradox, as well as testable hypotheses. In brief, I will argue that a single negative emotion such as sadness can be conceptualized according to the subjective feelings (SFs) it generates (one process), but also its aversive tendency (another process, operating in parallel). I will propose that in an aesthetic context, such as listening to music, these two processes can decouple, with aversive tendency inhibited, while still allowing negative feelings to be experienced. I call this the Parallel Processing Hypothesis (PPH).

## Component Process Theory of Emotion

While emotions can be conceptualized in terms of discrete elements, such as being sad, or happy, or angry and so on, another approach is to classify the components that go into making up any particular emotion. Russell (1980) and Schlosberg (1952), have proposed that any emotion can be described in terms of its negative-positive aspect, as well as an activity/arousal aspect. Another componential approach is to treat an emotion as a collection of synchronized processes that work together to form emotional concepts. In Scherer’s (Scherer, 1984, 2005; Scherer et al., 2006) seminal component process theory of emotion, emotion is defined as “an episode of interrelated, synchronized changes in the states of all or most of the five organismic subsystems in response to the evaluation of an external or internal stimulus event as relevant to major concerns of the organism” (e.g., Scherer, 2001, p. 93, Scherer, 2005, p. 697). The model consists of the five functions and corresponding components for each subsystem, namely: (1) Evaluation of objects and events—Cognitive component (appraisal); (2) System regulation—Neurophysiological component (bodily symptoms); (3) Preparation and direction of action—Motivational component (action tendencies); (4) Communication of reaction and behavioral intention—Motor expression component (facial and vocal expression); and (5) Monitoring or internal state and organism-environment interaction—SF component (emotional experience). These are summarized in Table 1.

**Table 1:** **Component process theory of emotion in relation to enjoyment of negative emotion in music**.

Component Label	Component	Subsystem function	Enjoyment of negative emotion example
1. Appraisal	Cognitive component	Evaluation of objects and events	Context detection/knowledge (music, aesthetic)
2. Bodily symptoms	Neurophysiological component	System regulation	Bodily symptoms of the negative emotion
3. Action tendencies	Motivational component	Preparation and direction of action	Inhibition of aversion/hostility tendencies
4. Facial and vocal expression	Motor expression component	Communication of reaction and behavioral intention	Facial symptoms of the negative emotion
5. Emotional experience	Subjective feeling component	Monitoring or internal state and organism-environment interaction	Subjective feeling of the negative emotion (fully fledged, real negative emotion)

*Note. The component label and component name is used interchangeably or in combination in this article for ease of reading. They are presented separately in the table to be consistent with Scherer. The fourth column shows what each process produces in the case of a negative emotion experience in response to a piece of music (i.e., in an aesthetic, non-utilitarian context), namely the decoupling or dissociation of the action tendency (component 3) from components 2, 4 and 5 via the appraisal component (1), which detects the aesthetic context*.

**Figure 1:**
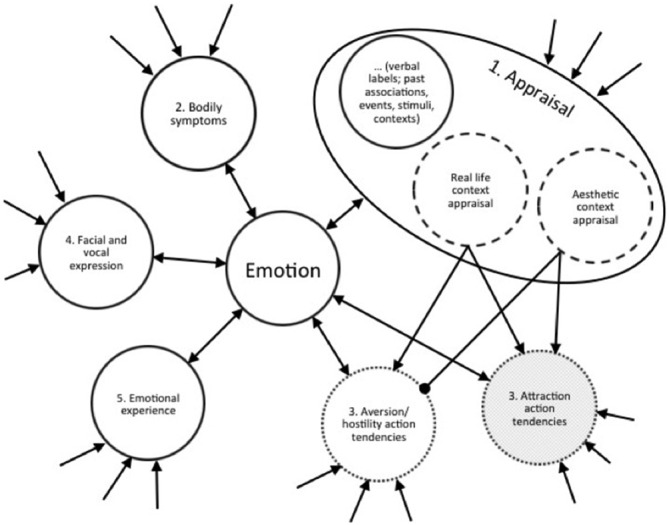
**Parallel processes network model of emotion.** The figure shows a portion of a “network” for a sample emotion, with the links around an emotion being the synchronized cluster of processes activated by the functions of the component process model of emotion. It also shows how context (one or other of the dashed circles, being part of the appraisal process 1) influences the nature of the action tendency (which will be one process from the two dotted circles that together control process 3). The emotion is part of a much larger network (as indicated by the incoming arrows). Aesthetic context inhibits aversion/hostility action tendency, indicated by the dot (not arrow) terminator. If the sample emotion is anger, or if the listener has low trait absorption, the aesthetic context appraisal will not form an inhibitory link to the aversion/hostility tendencies, contrary to the case indicated in the figure. However, if the emotion is sadness induced by music and the individual has high trait absorption, the aversion/hostility action tendency process is inhibited. A transmitting (non inhibited) arrow link does not assure that a process to which it connects will be activated, but simply enables activation. Verbal labels for the emotion, past autobiographical associations and so on are included in the Appraisal subsystem for convenience. But associations and dispositions could also be represented by external links. The attraction action tendency of process three may be redundant, hence gray shading (see text). The figure demonstrates the parallel processes model of emotion which is based on Scherer’s component process theory of emotion, and the layout is inspired by Bower’s (1981) associative network theory of memory and emotion.

The model can be adapted and applied to the problem at hand. It is hypothesized that the appraisal component (1) assesses, among other things, the context in which a stimulus appears. For now, I will limit context to two kinds: aesthetic (e.g., listening to music) and real life (those experienced in day to day life, including episodes of potential or actual distress, which Scherer, 2004 refers to as “utlilatarian”). The idea that context can manipulate the interpretation and experience of emotion has roots in the work of Schachter and Singer (1962). Their research demonstrated a dissociation between what they referred to as physiological and cognitive components of emotion: “It is the cognition which determines whether the state of physiological arousal will be labeled as “anger,” “joy,” “fear,” or whatever (p. 380). Scherer maintains this cognitive component in his model, and this component is to be understood as being the context detector. That is, in the present study context, via the cognitive appraisal component, determines whether an emotion is to be interpreted as having a positive or negative action tendency. The subjective emotional experience component is usually coupled with the motivational action tendency (component 3, in Table 1, being either negative [aversion/hostility] or positive [attraction]), but they do not have to be coupled. According to the hypothesis, the evaluation of an aesthetic context (via process 1) allows those emotions more susceptible to change in “direction of action” function (of component 3) to be dissociated. I will argue that sadness is one such emotion, although to date, there appear to be no explicit empirical accounts in the emotion literature of a dissociation between subjective emotional experience and action tendency based on context. This parallel processes interpretation is presented in Figure 1 in a form inspired by Bower’s (1981) associative network theory, but here the network is built around the five processes.

According to the PPH some emotions, such as sadness, are flexible in terms of their motivational component. In a real-life context sadness controversially has both a negative subjective feeling component (5) and an aversion motivational tendency (3). The valence is coupled. However according to the proposed explanation, in an aesthetic context the motivational tendency can be changed—it can have an approach tendency, or to be more precise the aversion/hostility motivation tendency is inhibited, even though the subjective feeling component (5) is still negative. As a contrasting example, an emotion such as annoyance might be more stable regardless of context (1) because it exhibits both aversive/hostile tendency (3) and negative subjective feeling (5). Evidence of which emotions might be coupled and which might not are discussed below. The rightmost column of Table 1 presents an example of how negative emotions and pleasure might occur simultaneously, with the appraisal component (1) being the gatekeeper of action tendency valence. Before discussing the evidence for this flexibility some definitions are required.

## Definitions

The study of sad music enjoyment is situated primarily in the fields of aesthetics and psychology. Aesthetics research involves many terms that have fluid definitions. Three terms of concern in this article require specific treatment in this respect: “aesthetic”, “non-aesthetic” (or “not aesthetic”), and “context”. Finally, action-tendency is defined as an indicator of preference.

“The contemplation of beauty” is a narrow definition of aesthetic that has roots in Alexander Baumgarten’s mid 18th-century appropriation of the term. Baumgarten was interested in understanding how the appropriate perception of beauty was a marker of good taste in one object over another (Shimamura, 2012). Today, taste is seen as a social construction (particularly since the work of Bourdieu, 1984/1979). Cognitive psychologists tend, instead, to use less loaded terms that lend themselves well to simple, self-reported rating, such as preference, pleasure or, more generally, hedonic tone (although these terms or not immune to cultural construction, either; see e.g., Masuda et al., 2008). Alternatively, a broader definition of “aesthetic” encapsulates both liked and disliked stimuli into the realm of aesthetic (e.g., Hargreaves, 1986; Palmer et al., 2013), as well as the contemplation of the beautiful, the ugly and any number of aspects of the inducing stimulus (Lee and Anstruther-Thomson, 1912; Adorno, 1997). Although the broader definition is adopted by some in the cognitive sciences, it is in part an artifact of anarchistic modernism and post-modernist ideals (for further discussion, see Shimamura, 2012). A definition that captures the spirit of aesthetic experience, while remaining conducive to systematic empirical investigation is “the *positive* hedonic toned response to a stimulus which brings with it no day-to-day utilitarian consequences” (see, e.g., Scherer, 2004). As Hoyer and Stokburger-Sauer (2012 p. 168) put it “[b]ecause aesthetics is considered as something positive that is somehow related to beauty, a positive valence is inherent in this term.” In short, in this article, aesthetic—and therefore musical—experience will be treated as a positive experience, inducing any or all of preference, enjoyment or liking in response to a piece of music.

Non-aesthetic experiences are those that attract utilitarian thoughts: that is, those which are focussed on real-life, day-to-day outcomes, such as doing the shopping, preparing for work, having a meal. However, in addition, but consistent with this definition, stimuli that are intensely disliked cannot be aesthetic. In contemplating or attempting to contemplate a hated piece of music, the individual is drawn away from, or never reaches, an aesthetic state because of the real-life annoyance that the stimulus generates. A piece of music can be disliked so much that it might be judged as “this just is not music to me, it is noise”.

An aesthetic “context” is defined as an environment (or more generally, a schema) which facilitates, or is associated with the contemplation of an aesthetic stimulus, such as a concert hall, an art gallery, a grand, historic cathedral, or an ipod. An aesthetic context requires both an aesthetic environment and an aesthetic (as distinct from non-aesthetic) stimulus to be perceived. One without the other will not generate an aesthetic context (for a detailed discussion, see Schubert et al., in press).

As described in the previous section, action tendency is a tendency to approach or avoid an object or environment. The *action*, when it occurs, is objectively observable (e.g., orienting or moving toward an object), making it a well-defined aspect of the phenomenon. However, the “tendency” aspect means that there is an internal, mental, more difficult-to-measure aspect (Berridge, 2003). Some simplification and compromise is expedient. Experiences described in terms of preference, pleasure and enjoyment are highly compatible with approach *tendency*, and displeasure/pain (annoyance, hatred, frustration etc.) is compatible with avoidance/hostility *tendency*. In other words, the motivational component, like the subjective feeling component, also contains mental content. It is not a cognitively empty vessel consisting solely of musculoskeletal motor events towards or away from the related object. Work by Rozin (1999), Elliot and Covington (2001), Vorderer et al. (2004), Frijda (2009) and Arnold and Reynolds (2012) suggests that positive action tendencies, on their own (e.g., even when not accompanied by positive subjective feelings), can be experienced as pleasurable.

## Evidence for the Parallel Processes Hypothesis (PPH)

Recent empirical evidence provides some support for PPH. In gathering data on hated and loved music, Schubert (2013b) found that negative emotions of anger, frustration and boredom were associated with hated music, while sadness was reported for both hated and loved music. That is, while sadness was evoked by music that is loved (invoking approach action tendency) *and* music that is hated (invoking avoidance/hostility action tendency), certain negative emotions appeared to be reserved for hated music only. This asymmetry suggests differential experiences within an emotion depending on context (hated music evokes a non-aesthetic, day-to-day context, while loved music may invoke an aesthetic context). Furthermore, it suggests that some emotions, such as sadness, may be experienced as sad regardless of the condition (hated music or loved music condition). The explanation that is being explored here is that something about the nature of the negative emotion changes across these conditions. The change is interpreted as being caused by context. PPH can explain these data. Compensation theories require the positive aspect of the aesthetic context to occur as a separate emotion.

Evidence from explicit, self-report research of action-subjective feeling decoupling as a result of context can be found in Taruffi and Koelsch (2014). They collected responses to a questionnaire in which participants rated various reasons for listening to sad music. In a stimulus free survey, participants were asked to consider music that expressed sadness. Using factor analysis, four dimensions of rewarding aspects of sad music were identified: (1) No “real life” Implications; (2) Emotion Regulation; (3) Imagination and (4) Empathy. The latent variable Emotion Regulation received a relatively high mean score, and this presents evidence for a compensation explanation for enjoyment of sad music because, for example, the sad music makes the listener feel better after listening. However, the highest mean score was for the variable No “real-life” Implications, suggesting that individuals are able to raise aesthetic/musical context appraisal to consciousness, and identify this as a reason that allows sad music to be liked. That is, the listener can enjoy negative emotion in music not necessarily because it is accompanied by something that is somehow positive. The sadness itself seems to be enjoyed, and the awareness that the sadness does not cause “actual” harm is possible because of the context being aesthetic, rather than a day-to-day, real-life event.

If one accepts this context dependent distinction within an emotion, then there is no paradox in the enjoyment of negative emotion. The feeling of sadness is active in parallel with the motivational tendency of attraction because of the detected aesthetic context. The case of sadness is perhaps the simplest to explain, but any negative emotion that is enjoyed in an aesthetic context can be explained by these parallel, yet interrelated, processes.

## Enjoyed Or Just Not Disliked?

In an aesthetic context a wide range of subjective feelings in process 5 (Table 1) can be experienced with the aversive/hostile tendencies inhibited. But this only explains why some negative emotions such as sadness might not induce aversive/hostile motivation tendency. It does not explain why one would enjoy the sadness evoked. The attraction motivation tendency provides one solution (showed in gray in Figure 1), but a more parsimonious solution was proposed by Martindale (1984, 1988): that the activation of mental representations is in and of itself pleasurable. And so, in the absence of aversive/hostile tendency, any kind of thought is enjoyable. The activation of different thoughts and feelings adds to the sum total of activation, and therefore the amount of pleasure generated, even while experiencing sadness. Components 1, 2, 4 and 5 all add to the amount of cognitive activation. It is only component 1 that can switch the interpretation of a stimulus/event such that process 3 inhibits aversion/hostile or it does not (for further discussion, see Schubert, 1996). The attraction motivation tendency of component 3 is redundant according to this view (hence grayed out in Figure 1). In other words, the net, scalar amount of activation of the other components feed into component 3.

## Individual Differences

The contextual aspect (real-life or aesthetic) is determined by some objective environmental factors (e.g., a concert auditorium is an environments that can evoke an aesthetic context), but also by the way the individual interprets the context. Hence, the attraction to sad music is dependent on factors such as mood, past associations and personality in addition to “objective” context. There exists evidence that certain individuals have a greater propensity to enjoy negative emotion in music than others. People who score high in empathic concern (Garrido and Schubert, 2010, 2011; Vuoskoski and Eerola, 2012; Vuoskoski et al., 2012), absorption (Garrido and Schubert, 2010, 2011, 2013, 2015) and openness to experience (Ladinig and Schellenberg, 2012; Vuoskoski et al., 2012) have a greater tendency to enjoy negative emotion in music. Young males also report greater enjoyment of negative emotion in music than other demographic groups (Chamorro-Premuzic et al., 2010; Eerola et al., 2015). PPH explains these differences by positing preferential activation of the aesthetic context and subsequent inhibition of negative action tendency in some individuals at certain times more than for others and at other times. For example, a person with low trait absorption, empathy and openness to experience may have more fixed, stable, context independent experiences of most emotions (especially negative emotions, such as sadness). On the other hand, those who enjoy nostalgic experiences, even if tinged with sadness, may do so because they have greater flexibility in their appraisal of context, while still being able to experience the subjective feeling component as a negative emotion.

## Testing the Parallel Processes Hypothesis

**Figure 2:**
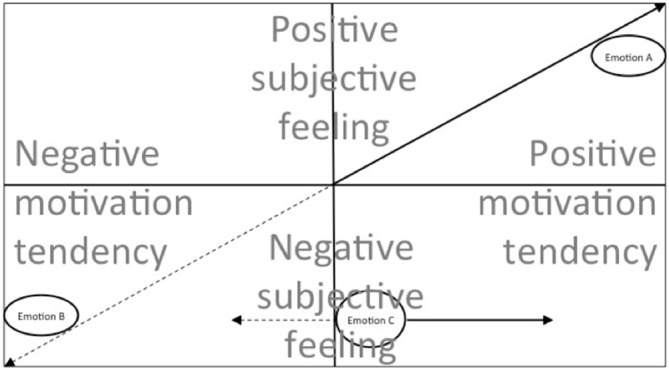
**Two component emotional valence space.** The space demonstrates how some felt emotions are coupled along the two dimensions, regardless of context, exemplified by sample emotions A and B, which could be happiness (positive subjective feeling (SF) and positive motivational tendency) and annoyance (negative SF and negative motivational tendency) respectively. However some emotions, such as C, are flexible along the motivational tendency component in that they can move along that component according to context. For example, if sample emotion C is sadness, in an aesthetic context it will not be in the negative motivational tendency region, but still retain its negative SF. The flexibility is mediated by individual differences (such as trait absorption) and context (aesthetic vs. real life). The motivational tendency component can be understood in a number of ways, including level of enjoyment, pleasantness, preference, attraction and liking. The SF component is related to emotional valence as shown in two dimensional (arousal, valence) emotion space (e.g., Russell, 1980), and is generally stable for any particular discrete emotion, regardless of context. If the model is correct, it will explain some of the confusion in understanding the apparent paradox of enjoying sadness in music. Compensation theories, for example, assume that the sadness of an emotion (e.g., at B) is subtracted from simultaneous happiness (e.g., at A), producing a lower residual negative output. Thus, for some researchers, motivation tendency and SF are coupled (treated as the same dimension). The present model suggests that they can be dissociated in some cases based on the context. Dotted line indicates negative motivation tendency. The region to the right consists of aesthetic emotions and real life emotions. The region to the left consists of real life emotions only. Aesthetic pleasure can be calculated not by summing the contribution along the SF component, as compensation theories do, but summing contributions along the positive motivation tendency. This explains how sadness can be experienced (negative SF), but at the same time adding to the pleasure (positive motivation tendency).

One way of falsifying PPH is to identify symmetric component pairings, namely negative emotions with hated music (a non-aesthetic context) and positive emotions with loved music (possibly an aesthetic context), and then demonstrate statistically no *positive* emotions in response to *hated* music and no *negative* emotions in response to *loved* music. Figure 2 outlines the hypothesis using a two component emotional valence space. The diagonal arrow falsifies the hypothesis, while the horizontal line supports it. Evidence of an exclusively diagonal locus of any emotion would indicate that the two components (feelings and motivation) are not dissociable, and PPH could be rejected. Furthermore, the dissociation might be mediated by individual differences, as mentioned above. In that case, the symmetry should be clearest for people with a low propensity to uncouple their motivational tendency process from their SF process, such as people low in trait absorption.

Some preliminary evidence in support of the hypothesis can be found in Schubert (2013b) where 28 negative emotion words were reported for loved pieces in comparison to only three positive emotion words for hated music. Low (statistically zero) counts in both of these cells would be needed to falsify PPH. This is in comparison to the positive-positive cell and the negative-negative cell in the same study, which drew a count of congruent valences that were 37 and 49 respectively. The marginal contribution of individual differences was not tested. It could be that the bulk of the 28 negative emotion words used to describe positive motivational tendency of loved music came from a particular personality type. Some further discussion of testing PPH is presented in the penultimate section of this article.

## Why Would Motivation and Subjective Feeling Become Uncoupled? The Role of Play

Why would dissociation between motivational and subjective feelings components occur? From an ontogenetic perspective play allows an individual to engage with a range of activities and skill development that are beneficial to future behaviors. Play facilitates practice of motor and cognitive processing (including social interaction). Suspension of the usual real-life motivation-subjective feeling coupling is desirable to allow exploration of a wide range of feelings under these circumstances. However, such an argument will raise the criticism that play, and therefore listening to music, must be functional and adaptive (Pellis et al., 2015). From the intrasubjective perspective, play is something in which one may engage simply for its own sake (van Leeuwen and Westwood, 2008). This is evident in one of the rare psychometric inventories designed to measure play experience in human adults. Pavlas et al. (2012) developed the scale which collects responses across four experiential dimensions labeled (1) freedom (sample item “The game gave me the freedom to act how I wanted to”); (2) no extrinsic (“I was not worried about someone judging how I performed in the game”); (3) play directed (“I was playing a game rather than working”); and (4) autotelic focus (“I wanted to do well in the game, ‘just because”’).

The freedom dimension shows how an individual may wish to be free to experience a wide range of emotions within the “safe”, make-believe setting of a game. And the other three dimensions are indicative of varying degrees of absorption in the activity. Music is also an activity in which cognitive experiences can be explored in a safe, “free” environment, while inviting the listener to become absorbed to various degrees. In this respect there is nothing particularly special about music. Like all the arts, it is a form of play (Walton, 1990; Schubert, 2009). One’s motivation to participate in music listening and other aesthetic experiences is similar to that of participation in many types of play. Importantly, such participation involves suspension of disbelief. Play endows us with the ability to decouple emotional feelings from their usual, real-life motivational tendencies.

## Other, Recent Parallel Processes Perspectives

Very few theories propose a parallel processes solution where the negative emotion itself can be interpreted as having some component that can simultaneously generate pleasure. Huron’s (2011) psychophysiological account asserts that the prolactin produced when crying (tears of sorrow, but not tears of joy) has positive effects such as analgesia and sensations of comfort. These effects are compensatory, bringing the organism back toward a level of homeostasis (internal balance). This alone does not generate pleasure, only a return toward a nominally neutral level. However, Huron (2011) proposed that since the listener is responding to the music, where there was no “real” reason to cry, the effect is of overcompensation, making the physiological change translate into a net pleasurable experience. While the mechanisms involved occur in parallel (sad feelings followed by prolactin release followed by the realization that it was aesthetic sadness), the *effect* can be interpreted as compensatory. The theory explained attraction to emotions that are associated with the production of prolactin, in particular sadness and grief, but not others.

Sachs et al. (2015) also offered a homeostasis account, arguing that people with particular personal traits use sadness in music to obtain satisfaction in different ways in response to differential environmental factors: to compensate for a distressing situation for an absorption prone individual, and to compensate for boredom for an individual with high openness to experience (see also Chamorro-Premuzic et al., 2010). These parallel processes theories are driven by a homeotstatic principle, and as such provide a good alternative against which the currently proposed cognitive processing approach can be compared experimentally. The critical point is whether the homeostasis mechanism is a more persuasive explanation than is context detection.

In a revised version of his influential theory of how emotions are communicated, Juslin (2013) proposed that two “mechanisms” interact to explain enjoyment of negative emotion: contagion and aesthetic judgment. “On this view, listeners *do* indeed experience “genuine” sadness—resulting from the Contagion mechanism—but they *also* experience pleasure which results from the perceived beauty of the music. If this hypothesis is correct, there is actually a “mixed” emotion of sadness and pleasure. It is not that the sadness *per se* is a source of pleasure, it only happens to occur *together* with a percept of beauty.” (Juslin, 2013, p. 258, italics in original). Juslin’s theory is therefore a compensation theory. There is no claim that sadness itself is pleasurable. Rather, for Juslin, pleasure occurs through an evaluation (the aesthetic judgment), in parallel with the sadness. Hence, Juslin’s (2013) theory does not consider the pleasure to be integrated with, or a component of, sadness itself. Furthermore, for Juslin the act of making an aesthetic judgment is at least to some extent a deliberate, volitional process: “the aesthetic judgment process begins with *an initial classification* of the music as “art”, which will lead the listener to adopt *an aesthetic attitude*. This means that the listener’s attention is focused on the music, and that aesthetic criteria are brought to bear on the music” (p. 247, italics in original). PPH on the other hand requires no explicit or implicit judgment for pleasure to be experienced. The implicit (aesthetic/music) context alone (the approximate equivalent of Juslin’s “classification as art”) is a sufficient initial condition of activating pleasurable response via the appraisal component.

These differences provide empirically testable alternatives. Juslin’s (2013) theory predicts that a positive aesthetic judgment must accompany sadness if the sad music is to be enjoyed (Juslin, 2013, p. 256; but this view is less emphatic in Juslin and Isaksson, 2014). And so, for Juslin (2013), there must be some other quality, namely an aesthetic judgment such as beauty, present in the music that explains why the sad music is enjoyed. Presumably, too, the listener must be conscious of this beauty (because it is a judgment). PPH, instead, predicts that sadness itself is sufficient in generating pleasure, provided the context is musical/aesthetic, and that the pleasure generated is implicit: it may occur without any explicit judgment pertaining to some causal quality of the music. One way of testing these alternative predictions is to manipulate context (music/aesthetic vs. everyday) and measure enjoyment and aesthetic judgment, if any, in response to sad music. According to PPH, once aesthetic context is detected, it should be possible to enjoy negative emotion without any necessary judgment or additional positive emotion. For PPH, context, controlled by the appraisal component, is the gatekeeper of pleasure, while for Juslin’s theory aesthetic judgment plays an important compensatory role. Juslin’s theory predicts that when the listener makes an aesthetic judgment the music will, usually, be enjoyed. Both perspectives predict that the presence of emotion can aid or embellish the preference, and both acknowledge the important role of culture in shaping and defining aesthetic experience (whether it be through context or through aesthetic judgment). Indeed, apart from (1) the compensation vs. parallel components; and (2) the deliberate aspect of aesthetic judgment vs. the implicit aspect of context detection, the two explanations are fairly similar. But there are three more differences that are worth noting: (3) position along the cognitive processing path; (4) ethological justification; and (5) explanation of other, non-sad negative emotions.

The two explanations occur at different points along cognitive processing path of a particular aesthetic experience. Apart from auditioning and perception, context must precede aesthetic judgment because without the appropriate context aesthetic judgment cannot occur. Consider a thought experiment. Suppose that the contagious experience of seeing a close friend feel sad can be calibrated to the same “amount” of subjective sadness as the piece of music that makes one sad. It is unlikely that the (real life, utilitarian) context of the friend’s sadness will be associated with beauty. The context (appraisal) must play a pivotal role (Schubert, 1996, 2009, 2010). And so, Juslin’s aesthetic judgment seems to be something that happens a bit further along the cognitive processing chain. The context must have already been detected for the judgment of beauty to be possible, should the judgment be made at all. In PPH, the role of the appraisal process in detecting context provides the gatekeeper of the action tendency valence (attraction vs. avoidance/hostility).

Furthemore, Juslin’s account does not at this stage present a possible ethological basis for the pleasure generated by “pleasurable sadness”: aesthetic judgment does (but sometimes does not) convert emotional experiences into pleasurable ones, but it is not clear why this should happen. PPH, on the other hand, has an ethologically plausible explanation, having origins in, but also being bounded to, play activities, as discussed above. Context detection differentiates between play activities, such as music perception, and real life, utilitarian activities; culture shapes the kinds of play activity that we refer to as music listening, as it does with watching a film or a play, or reading a novel or poem, or playing a computer game, or watching sport, as it does too with the creation of and participation in each of these. Juslin is aware of the role of context, but in the present approach, context is central.

Finally, Juslin, like some of the researchers cited in this article, prefers to contain the explanation to sadness, and not to other negative emotions. Some evidence exists that other negative emotions can be enjoyed (Huron, 2015; Schäfer et al., 2015; Sharman and Dingle, 2015), and the dismissal of such possibilities may be symptomatic of a general scepticism in the literature about whether music can evoke a range of negative emotions that can be enjoyed. The cause of this scepticism could be attributed to the regular classification of the subjective feeling being treated as the same semantic class as motivational tendency, since the two are so obviously correlated most of the time (as shown in Figure 2, see also Schubert et al., in press).

## Conclusion

This article has argued that attraction to negative emotion in music can be explained as parallel processes rather than as a paradox. These processes are activated by context, which is predisposed to personality, moods, past experiences, cultural conditioning and social norms in addition to environmental factors. The PPH has implications for the debate on whether it is possible to experience both positive and negative emotions simultaneously (Larsen et al., 2001; Hunter et al., 2008, 2010). The present account if considered worthwhile will demand a considerable program of empirical investigation.

## Author Contributions

Sole authored. The author confirms being the sole contributor of this work and approved it for publication.

## Funding

This work was supported by Grants from: Australian Research Council FT120100053.

## Conflict of Interest Statement

The author declares that the research was conducted in the absence of any commercial or financial relationships that could be construed as a potential conflict of interest.
